# Adolescent Cyberbullies’ Attributions: Longitudinal Linkages to Cyberbullying Perpetration

**DOI:** 10.3390/ijerph20126083

**Published:** 2023-06-08

**Authors:** Michelle F. Wright

**Affiliations:** Department of Psychology, DePaul University, Chicago, IL 60614, USA; mwrigh20@depaul.edu

**Keywords:** cyberbullying, attribution, adolescent, motivations

## Abstract

The aim of the present study was to examine cyberbullies’ attributions pertaining to their perpetration of cyberbullying, and how such attributions relate to their cyberbullying behaviors six months later. Participants were 216 adolescents (*M* = 13.46, *SD* = 0.62 years; 55% female) from the suburbs of a large Midwestern city in the United States. They were interviewed face-to-face in the fall of 2018 concerning why they acted in negative ways toward peers online or through text messages. They also answered questionnaires regarding how often they perpetrated face-to-face bullying and cyberbullying during the fall of 2018 and spring of 2019. The attributions of revenge, convenience, anger, and anonymity each predicted cyberbullying at the second time point while controlling for face-to-face bullying perpetration. Results from this study provide important information to the literature regarding cyberbullies’ attributions for perpetrating cyberbullying, and how such attributions predict future cyberbullying perpetration. These findings are important for the development of antibullying programs that might aim to change adolescents’ attributions for cyberbullying perpetration to reduce continued engagement in these behaviors.

## 1. Introduction

Research on the social cognitive (e.g., attributions) correlates of adolescents’ perpetration of cyberbullying has been slow to develop. Such investigations are important, as adolescents’ attributions relate to their engagement in both face-to-face bullying and cyberbullying perpetration [1]. Most of the investigations aimed at understanding the relationship between attributions and adolescents’ involvement in these behaviors include methodologies that ask adolescents to evaluate hypothetical situations. Follow-up examinations focused on adolescents’ attributions regarding their *actual* behaviors is a necessary next step in the field as the available research indicates that evaluations of real situations, rather than imagined, produce more valid results [2]. Utilizing the social information processing model [3] as a theoretical framework, the present study sought to understand cyberbullies’ attributions pertaining to their perpetration of cyberbullying, and how such attributions relate to their cyberbullying behaviors six months later.

### 1.1. Cyberbullying Perpetration

Defined as targeting someone with hostile, intimidating, threatening, and embarrassing behaviors using information and communication technologies (e.g., email, online games, gaming consoles, mobile devices, social media) [4], cyberbullying has been conceptualized by researchers as being an extension of traditional face-to-face bullying perpetration [5,6,7]. Like face-to-face bullying perpetration, cyberbullying behaviors involve an imbalance of power between victim and bully, while also including a technological component. The potential repetitiveness of cyberbullying also makes it like traditional face-to-face bullying, with technological mediums adding an additional level of complexity to cyberbullying behaviors. For example, a victim can be targeted with a humiliating and embarrassing video that can be sent to one person or a multitude of bystanders, who are also able to forward the content to yet more bystanders, with or without malicious intent. Examples of cyberbullying behaviors include rumor spreading, harassment, social exclusion, humiliation, physical threats, gossip, and verbal insults, as well as physical forms of cyberbullying, such as hacking. Other behaviors include making anonymous phone calls, forwarding explicit or embarrassing videos, identity theft, pretending to be someone else, and harassment via social networking websites and/or text messages [8,9].

Cyberbullying is related to an assortment of adjustment difficulties, including suicidal ideation, non-suicidal self-harm, depression, anxiety, subjective health complaints, and lower wellbeing. The negative consequences associated with cyberbullying have increased researchers’, educators’, and parents’ concerns with perpetration of these behaviors. Cyberbullying is also associated with lower academic performance, increased school absences, and greater truancy [7,10]. Another study found that greater absenteeism and lower grade point averages were associated with cyberbullying perpetration after controlling for face-to-face bullying perpetration [4]. Perpetrators are also likely to experience externalizing problems, such as drug use and violence [7,11,12]).

Not only are researchers concerned with cyberbullying because of the associated negative consequences, but they are also concerned with understanding the motivations surrounding adolescents’ perpetration of cyberbullying. Wright [13] and Ybarra et al. [4] proposed anonymity as a potential mechanism increasing the likelihood of perpetrating cyberbullying. The ability to hide or mask one’s identities through information and communications technology is associated positively with cyberbullying perpetration among adolescents [4,7,14]. Anonymous forms of cyberbullying also vary from study to study, with one researcher finding 12% of adolescents in her study perpetrated cyberbullying [15] and another researcher finding rates as high as 80% for anonymous perpetration of cyberbullying [11]. Forums designed to maintain anonymity also increase cyberbullying whereas forums that require less anonymity do not (Moore et al., 2012). In addition, a handful of studies have found that greater confidence with remaining anonymous using information and communication technologies increased anonymous cyberbullying perpetration [13]. Although anonymity is proposed as a factor contributing to cyberbullying perpetration, it is unclear whether adolescents indicate that such an ability dictates their behaviors. The studies reviewed in this section assessed beliefs about anonymity online, instead of asking adolescents *why* they perpetrated cyberbullying. Thus, a fruitful direction in understanding the perpetration of cyberbullying is to also investigate adolescents’ attributions.

### 1.2. Attributions

Attribution refers to the interpretation of cues and social behaviors [16]. According to social information processing theory, when adolescents encounter a social situation, they attend to situational and internal cues and then interpret the intent of others involved in the social situation [3]. Adolescents access their long-term memory for relevant information, while also engaging in a causal analysis to decide the intentionality of the person or people involved in the social situation. They compare their inferences to previous experiences with the person or people in their long-term memory. Decisions regarding the causes of behaviors influence adolescents’ thoughts and their subsequent behaviors. A lot of research has documented adolescents’ attributions for face-to-face bullying situations and have highlighted the attributions linked to later bullying and aggressive behaviors.

In the literature on attributions for traditional face-to-face bullying, researchers have found evidence supporting biased attributional patterns among adolescents who engaged in overt aggression and relational aggression. Findings from such studies suggest that aggressive adolescents make impulsive decisions about intentionality, assign hostile attributions (i.e., interpretation of benign or ambiguous behaviors as hostile) to ambiguous peer provocations, and use biased information to generate responses to various social situations, which increases further perpetration of bullying and aggression [17]. Furthermore, hostile attribution biases predict future aggressive behaviors, especially when perpetrators experience emotional distress [18]. Self-blame attributions are another type of attribution that applies to face-to-face bullying situations, and these attributions are often unrelated to future aggressive behaviors [19,20,21].

Although research has thoroughly documented the linkages between attributions and later bullying and aggressive behaviors, little attention has been given to how attributions regarding cyberbullying influence subsequent perpetration of these behaviors. The literature on this topic typically applies hostile attributions regarding face-to-face bullying and aggression to cyberbullying, with findings indicating that hostile attributions regarding face-to-face bullying are related to cyberbullying perpetration [22]. Findings from Wright’s [23] study indicated that aggressor-blame (i.e., attributing cyberbullying to the aggressor’s issues) and self-blame attributions (i.e., attribution cyberbullying to the victim’s issues) were both associated with cyberbullying perpetration six months later, while neutral attributions were negatively associated with cyberbullying perpetration. Wright’s study provided valuable knowledge to the literature on the attributions of adolescents for cyberbullying situations, but she did not consider *actual* attributions for perpetrating cyberbullying and adolescents’ ability to freely report attributional responses without promoting from closed-ended questions. Instead, Wright asked adolescents to rate various attributions for hypothetical forms of cyberbullying behaviors. In addition, self-blame attributions are often unlinked to perpetration of face-to-face bullying [19,20,21], although Wright [23] found that it was positively linked to later cyberbullying, but at a lower magnitude than hostile attributions. The discrepancy for the self-blame attribution might be attributed to hypothetical situations that occurred in the cyber context, and follow-up research is needed to better untangle adolescents’ attributions for cyberbullying perpetration. Focusing on perpetrators’ attribution, Graf et al. [24] found that cyberbullying perpetration was the result of recreation more so than offline bullying. Although the focus was on perpetration of cyberbullying, the study used questionnaires to assess attributions, which might have limited the possibility of spontaneous responses. Utilizing semi-structured interviews, Varjas et al. [25] found that high school cyberbullies indicated that they were internally motivated to perpetrate cyberbullying to redirect their feelings. Such a finding highlights the importance of examining attributions for cyberbullying utilizing semi-structured interviews to undercover the reasons for cyberbullying among adolescents.

It is important to expand the literature on attributions for cyberbullying perpetration because the cyber context might have unique features that separate it from similar behaviors in the offline environment. Face-to-face social situations often involve more social cues, which makes it easier for adolescents to identify intentionality, whereas online situations lack nonverbal and intentional cues that are usually present in the face-to-face context [26]. Research has revealed that attributions regarding cyberbullying typically are less hostile than those in the face-to-face context [27,28]. Oftentimes, adolescents interpret behaviors that might be classified as cyberbullying as not serious and as a joke [29]. Believing that cyberbullying behaviors might be a joke could indicate that aggressive interactions through information and communication technologies are considered normative by adolescents [30]. The problem with normalizing cyberbullying behaviors is that it could lead adolescents to believe that the behaviors are not serious and hurtful, which could increase their involvement in cyberbullying.

### 1.3. The Present Study

This study addresses a few gaps in the literature, including focusing on attributions for *actual* cyberbullying perpetration, incorporating a longitudinal design to investigate the associations between attributions and later cyberbullying perpetration, and allowing adolescents to provide their own unprompted report of their attributions for cyberbullying perpetration, utilizing semi-structured interviews. The purpose of the present study was to describe cyberbullies’ attributions for engaging in cyberbullying perpetration, and to investigate the relationships between adolescents’ attributions and their subsequent cyberbullying perpetration, six months later, after controlling for previous levels of face-to-face bullying and cyberbullying perpetration. The following research questions were addressed by this study:

1. What attributions do adolescent perpetrators of cyberbullying report for engaging in cyberbullying behaviors?
2. What, if any, association is there among adolescents’ attributions for cyberbullying perpetration and their cyberbullying perpetration, measured six months later and while accounting for previous face-to-face bullying and cyberbullying perpetration?

## 2. Methods

### 2.1. Participants

Participants were 216 adolescents (*M* = 13.46, *SD* = 0.62 years; 55% female) from the suburbs of a large Midwestern city in the United States. They were in the 8th grade and from one of six middle schools located in predominantly middle-class neighborhoods. Most adolescents self-identified as white (60%), followed by Latinx (23%), Black/African American (20%), Asian American (5%), and other (2%). The average family income of the neighborhoods surrounding the schools was USD 63,499/year. At the six middle schools, approximately 33% to 48% of students received free or reduced cost lunch.

### 2.2. Procedures

**Figure 1:**
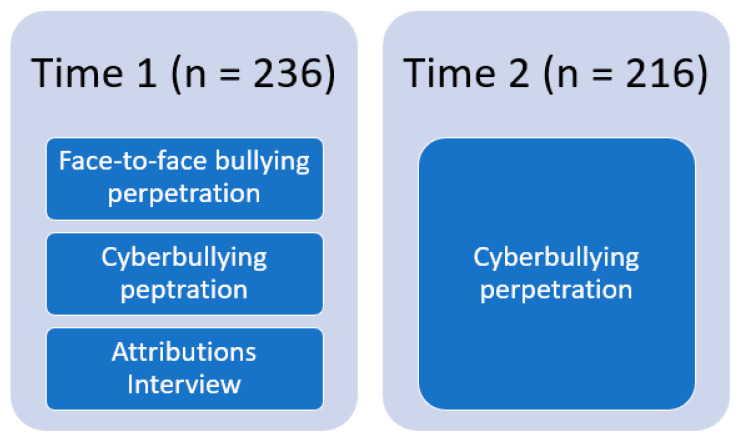
Flow of study activities from Time 1 to Time 2.

This study received ethical approval by the author’s university, and American Psychological Association standards were followed throughout the study’s duration. Middle schools were identified by creating a list of over 150 middle schools in the suburbs of the large Midwestern city. There were 20 schools randomly selected from the list and recruitment emails were sent to the school principals. Nine school principals agreed to allow their schools to participate in the larger study on cyberbullying experiences of middle school students. Meetings were conducted with school principals, teachers, and research personnel to discuss the purpose of the study and how adolescents would participate. After the meetings, classroom announcements were made, and parental permission slips were distributed to adolescents. Approximately 936 parental permission slips were distributed to 7th grade classrooms; of these, 888 parental permission slips were returned with a positive response, 23 parents/guardians declined to allow their child to participate, and the rest were unreturned. On the day of data collection, adolescents provided their assent to participate in the study. From the larger study, adolescents’ completed questionnaires regarding how often they engaged in cyberbullying; of these adolescents, 236 were classified as cyberbullies because their scores on the cyberbullying perpetration questionnaire were above the mean (*M* = 2.43, *SD* = 1.69), indicating that they had perpetrated cyberbullying at least every two months. These 236 adolescents were invited for a face-to-face interview at their school concerning why they acted negatively toward their peers online or through text messages at Time 1, during the fall of 2018 (see Figure 1 for a flow of activities at each time point). They completed questionnaires on their face-to-face bullying and cyberbullying perpetration as well. At Time 2, during the spring of 2019, adolescents completed the cyberbullying perpetration questionnaire. Twenty of the adolescents were not present for data collection at Time 2 because they had moved away or were unavailable for data collection.

### 2.3. Face-to-Face Interview

Semi-structured interviews were conducted face-to-face in a private location within the adolescents’ school. Trained research assistants performed the interviews with adolescents, which lasted between 10 and 15 minutes. Prior to the interviews, research assistants chatted with adolescents to increase their comfortability with the interview process and informed them about the privacy of their responses. At the beginning of the interview, research assistants asked adolescents about their online experiences and some of the “things” they did while online. Next, research assistants asked adolescents whether they ever acted in a mean or rude way online, and then asked the adolescent to elaborate. If the conversation did not involve cyberbullying, the research assistant asked the adolescent about this experience and whether they perpetrated it. After the adolescent indicated that they had engaged in cyberbullying at least once, they were asked the reason that they behaved the way they did. Adolescents were allowed to indicate all reasons as to why they engaged in cyberbullying perpetration. Transcripts of the interviews were recorded and then transcribed by a professional transcriptionist.

### 2.4. Coding

Two coders independently coded adolescents’ responses to the interview question regarding why they perpetrated cyberbullying. The coding scheme was adapted from previous research [31]. Wright et al. [31] provided a detailed description in their study regarding their coding scheme for adolescents’ attributions. Some modifications were made to the coding scheme by comparing Wright et al.’s participants’ responses to the adolescents’ responses in this study. This modification involved removing and adding categories based on adolescents’ responses. Initial codes were dummy coded, with “1” applying to a particular response and “0” if the response did not apply to a category. Multiple codes were assigned when adolescents’ responses reflected multiple categories. Afterward, codes with similar meaning were combined into categories based on theoretical considerations, and then later recoded into dummy variables of 0s (absent) and 1s (present). Disagreement between the two coders was resolved through face-to-face discussion. Kappas were between 0.81 and 0.90 for the coding of attributions, suggesting adequate reliabilities [32] and acceptable standards for previous studies on similar topics [33]. An 18-category coding scheme based on adolescents’ responses was developed (see Table 1). Categories included: revenge, convenience, dislike the victim, anger, jealousy, anonymity, justice, acceptance, prestige, displaced aggression, upset feeling, personal characteristics, self-esteem, insecurities, boredom, attention, conflict, and normative.

### 2.5. Measures

**Face-to-face bullying perpetration.** Ten items assessed adolescents’ perpetration of face-to-face bullying [34]. Sample items included: insult others to their face, threaten other peers, and turn others against a peer when they made me mad. The items were rated on a scale of 1 (*never*) to 9 (*daily*). This questionnaire was administered at Time 1 only, with Cronbach’s alpha of 0.89.

**Cyberbullying perpetration.** This measure is similar to the face-to-face bullying perpetration measure, except that the ten items asked adolescents how often they perpetrated cyberbullying behaviors [34]. Sample items included: insult others online or through text messages, threaten other peers online or through text message, and turn others against a peer online or through text messages when they made me mad. Items were rated on a scale of 1 (*never*) to 9 (*daily*). This questionnaire was administered at Time 1 and Time 2. Cronbach’s alphas were 0.86 for Time 1 and 0.88 for Time 2.

### 2.6. Analytic Plan

The sample size was chosen to detect small to medium effect sizes, using G*Power, provided by the University of Dusseldorf in Germany. Using *α* ≤ 0.05 and power (1 − *β*) of 0.80, a minimum sample size of 163 was needed. Descriptive statistics were conducted on the attribution categories to identify the frequency of each category and the raw number of times a particular category was identified. Correlation analyses were conducted among Time 1 and Time 2 face-to-face bullying perpetration and Time 1 and Time 2 cyberbullying perpetration. To test this study’s research questions, six hierarchical multiple regression analyses were conducted. Analyses were only performed for attribution categories reported by over 10% of the adolescents. Continuous predictors (i.e., Time 1 face-to-face bullying and cyberbullying perpetration) were centered. Block 1 included gender, 7th grade cyberbullying perpetration (Time 1), and 7th grade face-to-face bullying perpetration (Time 1). Block 2 included the attribution. Block 3 included a two-way interaction between gender and the attribution. Two-way interactions were tested using the Interaction program. This program provides the significance of the unstandardized simple regression slopes and provides graphical illustration of the simple slopes. Only those adolescents who participated in the study in Time 1 and Time 2 were included in the analyses.

## 3. Results

### 3.1. Descriptive Statistics

Correlations were performed between all Time 1 and Time 2 face-to-face bullying and cyberbullying perpetration questionnaires. Time 1 face-to-face bullying perpetration was related positively to Time 2 face-to-face bullying perpetration (*r* = 0.56, *p* < 0.001) and Time 1 (*r* = 0.33, *p* < 0.001) and Time 2 cyberbullying perpetration (*r* = 0.36, *p* < 0.001). Time 1 cyberbullying perpetration was related positively to Time 2 cyberbullying perpetration (*r* = 0.51, *p* < 0.001) and Time 1 (*r* = 0.28, *p* < 0.01) and Time 2 face-to-face bullying perpetration (*r* = 0.30, *p* < 0.001). A full correlation matrix of all coding categories, and Time 1 and Time 2 face-to-face bullying and cyberbullying perpetration is available upon request.

The most frequently reported attribution was revenge (44%), with one adolescent indicating that they “did me wrong first” as a response for cyberbullying perpetration. Convenience (e.g., “Easier than in real-life”) was reported by 29% of adolescents as to why they perpetrated cyberbullying. Dislike of the victim (e.g., “Do not care about him/her) was another reason and it was reported by 24% of adolescents. The attributions of anger (e.g., “I was angry about something that happened”) and anonymity (e.g., “I’m able to hide my identity online”) were reported by 17% of the participants for each attribution. Jealousy was reported by 13% of adolescents. The rest of the attribution categories were reported under 10% of the time from adolescents, including justice (e.g., “Wronged by this person and so I need to correct them”; 6.9%), acceptance (e.g., “Make myself look better”; 1.4%), prestige (e.g., “Make myself more powerful”; 1.9%), displaced aggression (e.g., “Bring others down to make myself feel better”; 9.3%), upset (e.g., “Upset over something else”; 7.8%), personal characteristics (e.g., “Like to hurt others”; 1.9%), self-esteem (e.g., “Want to boost my self-esteem”; 1.9%), insecurities (e.g., “I was feeling insecure that day; 3.2%), attention (e.g., “Want to get noticed”; 1.4%), conflict (e.g., “Angry at each other”; 5.6%), and normativity (e.g., “This is how everyone acts”; 0.5%).

### 3.2. Regression Analyses

The results of the multiple regression analyses are displayed in Table 2 for the attributions of revenge, dislike the victim, convenience, anger, anonymity, and jealousy. Gender was unrelated to Time 2 cyberbullying perpetration; 7th grade face-to-face bullying perpetration (Time 1) and cyberbullying perpetration (Time 1) were each positive predictors of Time 2 cyberbullying perpetration. The attributions of revenge, convenience, anger, and anonymity each were positive predictors of cyberbullying at Time 2. The convenience and anonymity attributions were unrelated to Time 2 cyberbullying perpetration, after controlling for gender, Time 1 face-to-face bullying perpetration, and Time 1 cyberbullying perpetration. A supplemental regression analysis with all attributions and interactions between attributions and gender is available upon request.

## 4. Discussion

Many investigations of the attributions for why cyberbullying perpetrators engaged in harmful behaviors do not consider *actual* behaviors, do not incorporate longitudinal designs, and typically utilize forced-response questionnaires to assess attributions. The present study utilizes the social information processing model as a theoretical framework and provides a methodological improvement over previous designs by describing perpetrators’ attributions for engaging in actual cyberbullying behavior and examining the associations between attributions and later cyberbullying perpetration, measured six months later. Findings from this study indicate that adolescents have many similarities in their reported attributions and that some attributions predicted later cyberbullying perpetration.

*Revenge* was the most frequently reported attribution. When asked why adolescents acted meanly toward a peer through text messages, one adolescent explained, “She said something mean to me first, so I was just getting her back.” The attribution of anger was reported by cyberbullies as well, and revenge and anger often occur together when it involves the motivation for engaging in cyberbullying. These findings are consistent with previous research indicating that anger and revenge are often motivators for cyberbullying [35]. Other attributions revealed that cyberbullies were motivated by the *convenience* and *anonymity* of the cyber context. These adolescents reported that cyberbullying was easier than bullying in the face-to-face context as it was the fastest way to spread information or to say something without actually saying it. Such findings are supported by the online disinhibition effect, which suggests that social restrictions and inhibitions are loosened over the Internet in comparison to normal face-to-face interactions, leading to more disinhibited aggressive behaviors [36]. The attribution of *dislike the victim* was reported by cyberbullies. Another attribution was *jealousy.* “I was jealous because a boy I liked asked someone else out,” said one adolescent. Some adolescents explained that they were motivated by jealousy, but they did not elaborate on any reason for their jealousy. These findings are further supported by previous research indicating that adolescents perpetrated cyberbullying because they disliked or were jealous of the victim [34].

The other attributions were less frequently reported by adolescents. The attribution of *justice* was interesting and was reported when adolescents believed they were wronged by the victim of their cyberbullying perpetration. Although such an attribution might fall under the umbrella of “revenge”, it implies that the victim clearly harmed the perpetrator in some way prior to cyberbullying perpetration and the perpetrator made it known that they were engaging in the behavior as a means of correcting the person’s previous actions. This attribution implies more of a social justice stance than revenge, although revenge is a very common attribution reported by adolescents in this study and in previous research [35]. Furthermore, online disinhibition might make correcting someone in the online context more salient and easier than in the face-to-face context [36]. *Displaced aggression* and *upset* attributions are slightly separate from the revenge attribution. These attributions indicate that the adolescent was wronged or upset by something else and then perpetrated cyberbullying toward someone *unrelated* to this previous harm. There is evidence that displaced aggression or being upset over something else occurs in the online context and can lead to cyberbullying behaviors [31]. The attributions discussed so far are slightly different from the *conflict* attribution. This attribution implies a mutual conflict between the perpetrator and victim. The conflict attribution was reported in a previous study on adolescents’ attributions for hypothetical relational aggression [34], although fewer adolescents reported this attribution when compared to what Wright and colleagues found.

*Acceptance, prestige*, and *attention* attributions seem to imply that the adolescent perpetrates cyberbullying to increase or promote their social standing in the peer group. During adolescence, adolescents are greatly concerned with their peers and many actively pursue a higher peer status [15], and therefore it might make sense that adolescents could perpetrate cyberbullying as a means of increasing peer status. The research literature supports the relationship between cyberbullying perpetration and higher social status among adolescents [15], providing additional support for the utilization of attributions related to status. The *personal characteristics* and *self-esteem* categories are attributions, like the previous described status-based attributions (i.e., acceptance, prestige, attention), which indicate that the adolescent is engaging in cyberbullying because of something about themselves. It is interesting that adolescents recognized that something within themselves made them desire hurting others online. It is unclear whether the personal characteristics and self-esteem attributions are typical attributions adolescents might make for engaging in cyberbullying perpetration. The desire to harm others and increase one’s self-esteem because one desires to do so seems like a hallmark of the dark triad of personality, as adolescents with these traits often are ready to exploit others and feel little remorse for doing so [37].

The least frequently occurring attribution was *normativity*. Runions et al. [30] indicated that believing cyberbullying is a joke could increase adolescents’ beliefs that such behaviors are normative and subsequently increase their engagement in cyberbullying. However, we found that very rarely did perpetrators of cyberbullying in our study report that the normativity of cyberbullying was a motivation. Although such behavior might be considered normative, it might be that it is often not the sole motivator for cyberbullying perpetration. 

To address research question two, we found that the attributions of revenge, convenience, anger, and anonymity each predicted cyberbullying perpetration at Time 2, while controlling for previous cyberbullying and face-to-face bullying perpetration. Due to the lack of research focused on which attributions are linked to cyberbullying, it is difficult to reconcile the current study’s findings with the literature. The available literature suggests that bullies of face-to-face aggression typically make more plans for revenge and that their patterns of bullying continue into adolescence [38,39]. Therefore, it is reasonable that their greater likelihood to seek revenge may continue their pattern of bullying. In addition, a variety of the literature links anger to bullying perpetration [40]. Attributions concerning the convenience and anonymity of the cyber context are consistent with previous research linking these variables to cyber aggression six months later [41]. Thus, finding that the attributions of revenge, convenience, anger, and anonymity predicted later cyberbullying perpetration appears to be consistent with the limited literature on this topic.

### Limitations and Future Directions

The sample size of adolescent perpetrators of cyberbullying was small, and thus it might make it difficult to generalize the findings to other groups of adolescents. Future research should aim to recruit a larger sample of cyberbullying perpetrators and interview them regarding their attributions for cyberbullying. This study also focused on adolescents, and there is increasing evidence that cyberbullying can occur as young as age nine. Therefore, longitudinal designs should be conducted with younger children and then follow them into adolescence to investigate whether attributions change over time and if such changes might impact cyberbullying perpetration. The assessment of cyberbullying perpetration was by self-report only. Follow-up studies should include a multi-informant approach to diminish the biases associated with self-reports. Peer nominations of cyberbullying perpetration have been performed in the previous literature and revealed adequate reliability and validity [34].

## 5. Conclusions

Adolescents’ attributions regarding cyberbullying perpetration might become internalized and incorporated within their mental database of their social interactions. They rely on their knowledge to process, understand, and respond to social information occurring in the cyber context. Results from this study have direct implications for intervention and prevention efforts aimed at reducing cyberbullying. Specifically, these findings inform the development of preventative intervention programs aimed at changing adolescents’ attributions regarding cyberbullying as such efforts may help to reduce these behaviors.

## Figures and Tables

**Table 1:** Coding scheme for adolescents’ attributions.

Coding Name	Example	Frequency and Percentages (*N* = 216)
Revenge	Get back at them. Did me wrong first.	95, 44%
Convenience	Easier than in real-life. Don’t want to say it to their face.	62, 29%
Dislike the Victim	Do not care about him/her. Dislike him/her. Hate him/her.	52, 24%
Anger	I was angry about something that happened.	36, 17%
Jealousy	I was feeling jealous.	28, 13%
Anonymity	Being anonymous makes it easier. I’m able to hide my identity.	36, 17%
Justice	Wronged or cheated by this person to teach them a lesson.	15, 6.9%
Acceptance	Make myself look better.	3, 1.4%
Prestige	Make myself feel powerful. Make myself feel important.	4, 1.9%
Displaced Aggression	Make myself feel better. Bring others down to make myself feel better.	20, 9.3%
Upset	Upset over something else.	17, 7.8%
Personal Characteristics	Like to hurt others. Want to make others cry. Want to embarrass others.	4, 1.9%
Self-Esteem	Want to boost my self-esteem.	4, 1.9%
Insecurities	I was feeling insecure that day.	7, 3.2%
Boredom	Nothing better to do. Bored with my life.	10, 4.6%
Attention	Want attention. Want to get noticed.	3, 1.4%
Conflict	In a conflict with that person. Angry at each other.	12, 5.6%
Normativity	This is how everyone acts.	1, 0.5%

Note. Some participants received multiple codes.

**Table 2:** Predicting cyberbullying from attributions.

	8th Grade Cyberbullying		
	β	*R* ^2^	Δ*R*^2^
Block 1		0.04	0.04 **
Gender	0.02		
7th grade cyberbullying	0.15 ***		
7th grade face-to-face bullying	0.04 **		
Block 2		0.60	0.55 ***
Revenge	0.30 ***		
Block 3		0.60	0.55
Gender × revenge	0.01		
Block 1		0.04	0.04 **
Gender	0.02		
7th grade cyberbullying	0.15 ***		
7th grade face-to-face bullying	0.04 **		
Block 2		0.66	0.63 ***
Convenience	0.27 **		
Block 3		0.66	0.01
Gender × convenience	−0.02		
Block 1		0.04	0.04 **
Gender	0.02		
7th grade cyberbullying	0.15 ***		
7th grade face-to-face bullying	0.04 **		
Block 2		0.03	0.01
Dislike the victim	0.07		
Block 3		0.04	0.01
Gender × dislike the Victim	−0.03		
Block 1		0.04	0.04 **
Gender	0.02		
7th grade cyberbullying	0.15 ***		
7th grade face-to-face bullying	0.04 **		
Block 2		0.01	0.01
Jealousy	−0.01		
Block 3		0.01	0.01
Gender × jealousy	−0.02		
Block 1		0.04	0.04 **
Gender	0.02		
7th grade cyberbullying	0.15 ***		
7th grade face-to-face bullying	0.06 *		
Block 2		0.66	0.61 ***
Anger	0.32 ***		
Block 3		0.66	0.01
Gender × anger	0.02		
Block 1		0.04	0.04 **
Gender	0.02		
7th grade cyberbullying	0.15 ***		
7th grade face-to-face bullying	0.03 *		
Block 2		0.66	0.63 ***
Anonymity	0.27 ***		
Block 3		0.01	0.01
Gender × anonymity	0.03		

* *p* < 0.05. ** *p* < 0.01. *** *p* < 0.001.

## Data Availability

Data is not publicly available due to privacy and ethical restrictions.
